# I-PfoP3I: A Novel Nicking HNH Homing Endonuclease Encoded in the Group I Intron of the DNA Polymerase Gene in *Phormidium foveolarum* Phage Pf-WMP3

**DOI:** 10.1371/journal.pone.0043738

**Published:** 2012-08-27

**Authors:** Shuanglei Kong, Xinyao Liu, Liwen Fu, Xiangchun Yu, Chengcai An

**Affiliations:** State Key Laboratory of Protein and Plant Gene Research, College of Life Sciences, Peking University, Beijing, China; New England Biolabs, Inc., United States of America

## Abstract

Homing endonucleases encoded in a group I self-splicing intron in a protein-coding gene in cyanophage genomes have not been reported, apart from some free-standing homing edonucleases. In this study, a nicking DNA endonuclease, I-PfoP3I, encoded in a group IA2 intron in the DNA polymerase gene of a T7-like cyanophage Pf-WMP3, which infects the freshwater cyanobacterium *Phormidium foveolarum* is described. The Pf-WMP3 intron splices efficiently *in vivo* and self-splices *in vitro* simultaneously during transcription. I-PfoP3I belongs to the HNH family with an unconventional C-terminal HNH motif. I-PfoP3I nicks the intron-minus Pf-WMP3 DNA polymerase gene more efficiently than the Pf-WMP4 DNA polymerase gene that lacks any intervening sequence *in vitro*, indicating the variable capacity of I-PfoP3I. I-PfoP3I cleaves 4 nt upstream of the intron insertion site on the coding strand of EXON 1 on both intron-minus Pf-WMP3 and Pf-WMP4 DNA polymerase genes. Using an *in vitro* cleavage assay and scanning deletion mutants of the intronless target site, the minimal recognition site was determined to be a 14 bp region downstream of the cut site. I-PfoP3I requires Mg^2+^, Ca^2+^ or Mn^2+^ for nicking activity. Phylogenetic analysis suggests that the intron and homing endonuclease gene elements might be inserted in Pf-WMP3 genome individually after differentiation from Pf-WMP4. To our knowledge, this is the first report of the presence of a group I self-splicing intron encoding a functional homing endonuclease in a protein-coding gene in a cyanophage genome.

## Introduction

Group I introns are self-splicing RNA sequences that are inserted into genes of a diverse range of bacteriophages of gram-negative bacteria, gram-positive bacteria and cyanobacteria. Most introns have been encountered in phages of *Myoviridae* or *Siphoviridae* family such as *Escherichia coli* phage T4 [1], *Bacillus subtilis* phage SPO1 [2], marine cyanomyovirus S-PM 2 [3], [4], [5] or *Xanthomonas Campestris* phage phiL7 [6]. Although the first description of group I introns in T7-like enteric bacteria phages ΦI and W31 (*Podoviridae* family) in 2004 [7], group I introns in T7-like phages have not been widely reported since, especially in T7-like cyanobacteria phages.

Many group I introns contain reading frames encoding a homing endonuclease gene (HEG) which is described as selfish genetic element [8]. Homing endonucleases (HEases) cleave single (nick) or double (DSB) strands at or close to the intron insertion site (IIS), generating strand breaks in homologous alleles that lack the intervening sequence (IVS). Subsequently, the strand breaks are repaired by homologous recombination using the allele that contains the HEG as a template [9]. As a result, group I introns are transferred into a new site [10]. However, different from typical intron-encoded HEases, I-HmuI and I-HmuII can cleave both intron-plus and intronless versions of their cognate genes. These two nicking HEases are encoded in group I introns in the DNA polymerase genes of *B. subtilis* phages SPO1 and SP82 [11].

HEases are divided into five families based on conserved nuclease active-site core motifs, catalytic mechanisms, biological distributions and wider relationships to non-homing nuclease systems. They are LAGLIDADG, HNH, His-Cys box, GIY-YIG and PD-(D/E)-XK motif in one HEase I-Ssp6803I [12], [13], [14]. HEases recognize extremely specific target sites spanning 14–40 bp. This means that cleavage by HEases is rare, making them possible to be used in genome engineering and gene therapy by highly efficient gene targeting in mammalian cells [15], [16]. However, HEases are tolerant to a variety of sequence variations within the recognition sequences [9].

**Figure 1:**
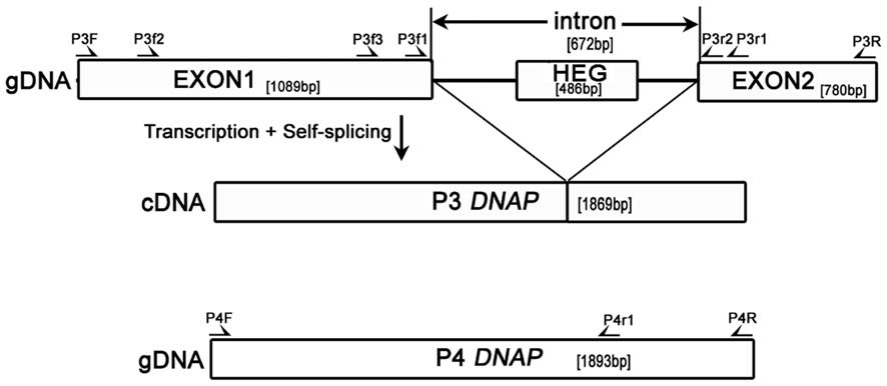
Schematic diagram of Pf-WMP3 and Pf-WMP4 *DNAP* genes. A HNH HEase is encoded in the 672-bp intron in the Pf-WMP3 *DNAP* gene compared to Pf-WMP4 which does not contain any IVS. The half arrows indicate locations of oligonucleotides used.

Pf-WMP3 and Pf-WMP4 are two closely related T7-like cyanophages which infect the freshwater cyanobacterium *P. foveolarum* and were isolated from Lake Weiming [17], [18]. In this article, we report the identification of a group I intron in the DNA polymerase (*DNAP*) gene of Pf-WMP3. This intron was initially found by DNA sequencing as an IVS (Figure 1). The intron was spliced *in vivo* and *in vitro* and inhibited growth when expressed in *E. coli*. A fully functional HEase (denoted I-PfoP3I) of the HNH family is encoded in this intron. I-PfoP3I nicked the intron-minus Pf-WMP3 *DNAP* gene and the Pf-WMP4 *DNAP* gene which did not contain any interfering sequence, indicating the variable capacity of I-PfoP3I. The recognition site of I-PfoP3I covers base pairs 2 to 15 downstream of the cut site on the coding strand. The endonuclease activity of I-PfoP3I purified from *E. coli* was independent of any added divalent cations, but apo-I-PfoP3I (I-PfoP3I without metal co-factors) required one of the metal ions, Mg^2+^, Ca^2+^ or Mn^2+^ to resume the endonuclease activity. Phylogenetic analysis suggests that the intron and the HEG elements might be inserted in Pf-WMP3 genome individually after differentiation from Pf-WMP4.

## Results

### *In vivo* Splicing of the Group I Intron in Pf-WMP3 *DNAP* Gene

**Figure 2:**
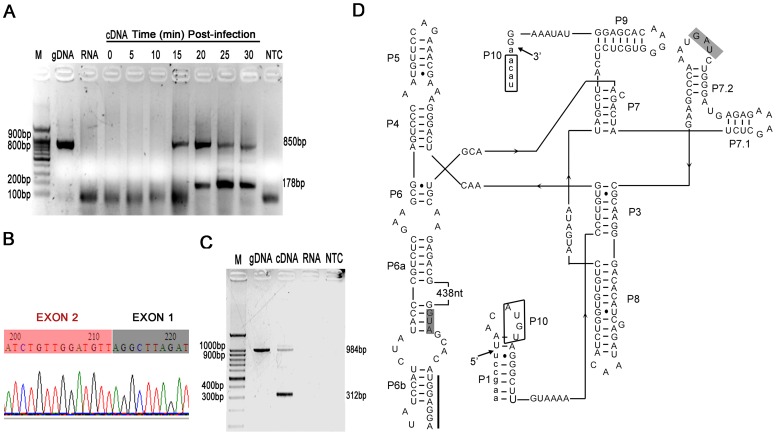
Splicing activity and predicted secondary structure of the group I intron of Pf-WMP3. A. *In vivo* splicing of the Pf-WMP3 intron. RNA isolated from Pf-WMP3-infected *P. foveolarum* was analyzed by reverse transcription. PCR was done using intron primers P3f1 and P3r1 on genomic DNA (gDNA), RNA before reverse transcription (RNA), cDNA and a no-template control (NTC). The times of RNA isolation (in minutes) after infection are indicated. An ethidium bromide-stained agarose gel (2% agarose [wt vol^−1^]) is shown. The sizes (in bases) of prominent bands of the DNA size standard (lane M) are indicated on the left. B. Determination of the splicing point by DNA sequencing of RT-PCR products of *in vivo* spliced transcripts. T7 primer in pEASY-T1 was used. C. *In vitro* splicing analysis of the Pf-WMP3 intron. PCR was done using intron primers P3f3 and P3r2 on genomic DNA (gDNA), cDNA (RNA was from *in vitro* transcription), *in vitro*-transcribed RNA and a no-template control (NTC). An ethidium bromide-stained agarose gel (2% agarose [wt vol^−1^]) is shown. The sizes (in bases) of prominent bands of the DNA size standard (lane M) are indicated on the left. D. Predicted secondary structure of the group I intron of Pf-WMP3. Exon and intron sequences are in lower and upper case letters respectively. Filled arrows indicate 5′ and 3′ splice sites. Conserved structural elements P1 through P9 are shown. Boxed regions designate the P10 pairing. Gray boxes indicate the start and stop codons of the I-PfoP3I coding sequence. The line next to AGGAGGU in the stem P6b indicates the putative ribosomal binding site (RBS). The 438-nucleotide sequence of the intron-encoded ORF is indicated.

The intron of Pf-WMP3 *DNAP* gene was tested for *in vivo* splicing activity from the primary transcript by nonquantitative RT-PCR. P3f1 and P3r1 specific primers gave products of 850 bp from genomic DNA (Figure 1) and unspliced transcripts (Figure 2A, lane gDNA) and 178 bp from the spliced transcript (Figure 2A, lane cDNA). From Figure 2A, we can observe that RNA isolated from cells presented both unspliced and spliced *DNAP* mRNAs. Additionally, RT-PCR amplified product was cloned into pEASY-T1 cloning vector (TRANS) and sequenced (Figure 2B). It was confirmed that the predicted location of this intron was correct by comparison with *DNAP* gene of Pf-WMP3 (Figure S1). There is no inverted repeat in the terminal regions of the intron sequence, suggesting that the IVS is not an insertion element or a transposon [19]. The IVS was also shown to be removable from the Pf-WMP3 *DNAP* precursor mRNA that was being translated in *E. coli* (BL21/pET*P3DNAP[+int]*, data not shown).

### *In vitro* Splicing of the Group I Intron in Pf-WMP3 *DNAP* Gene

To examine whether the intron can be excised *in vitro* from the primary transcript, intron sequence including flanking exons was cloned into pET-28a(+) in a proper orientation. The *DNAP[+int]* gene was under the control of the T7 promoter. RNA was obtained using T7 RNA polymerase from XhoI linearized plasmid pET*P3DNAP[+int]* (Materials and methods). This produced an RNA transcript with a 709 base 5′ exon, 672 base intron and 780 base 3′ exon. PCR was done using cDNA (from RNA generated by *in vitro* transcription) with P3f3 and P3r2 primers that recognized the flanking exons (Figure 1). The PCR product was 312 bp in size, corresponding to the size of the ligated exons (Figure 2C). This result indicated that the intron was able to self-splice *in vitro* simultaneously during transcription [20], [21]. Additionally, sequencing of the 312-bp PCR product confirmed correct exon ligation (data not shown).

### Prediction of the Secondary Structure of the Intron

Group I introns share conserved secondary structure elements which are necessary for ribozyme activity [22]. The secondary structure of Pf-WMP3 intron was predicted by Mfold program [23] with manual correction according to conventions for group I introns. The introns of ΦI and W31 [7] were referred to as models (Figure 2D). It folded into a typical group I intron structure with all conserved stem-loops P1 through P10 except P2. Secondary structure and the characteristic helical elements P7.1 and P7.2 linked by a G-U-A sequence in the intron assign it to subgroup IA2 [7], [24]. The conserved secondary structure elements are necessary for proper folding and excision. The open reading frame (ORF) is predicted to start in the P6a region and to span the P7 region with 486 bp, contributing to key structure elements of P6a, P6, P7, P7.1 and P7.2. The intron has a 4-bp long P10 paring between the sequence around the start of 3′ exon and a sequence near the 5′ end of the intron, promoting an alignment between the 3′ and 5′ splicing sites required for the ligation of exons. The characteristic sequence elements with a terminal exonic uracil at the 5′ spliced position forming a pair with guanosine in the P1 stem and a guanosine at the 3′ end are typical in most group I introns.

The intron also has a typical ribosome binding site (RBS), located 6 to 12 bp upstream of the start codon. Like introns in *DNAP* genes of T7-like bacteriophages ΦI and W31 [7], the RBS of Pf-WMP3 intron may reduce overall expression of the HEase compared with the product DNAP (in whose transcript the HEG is embedded).

### The Pf-WMP3 Intron Retards the Growth Rate of *E. coli*

**Figure 3:**
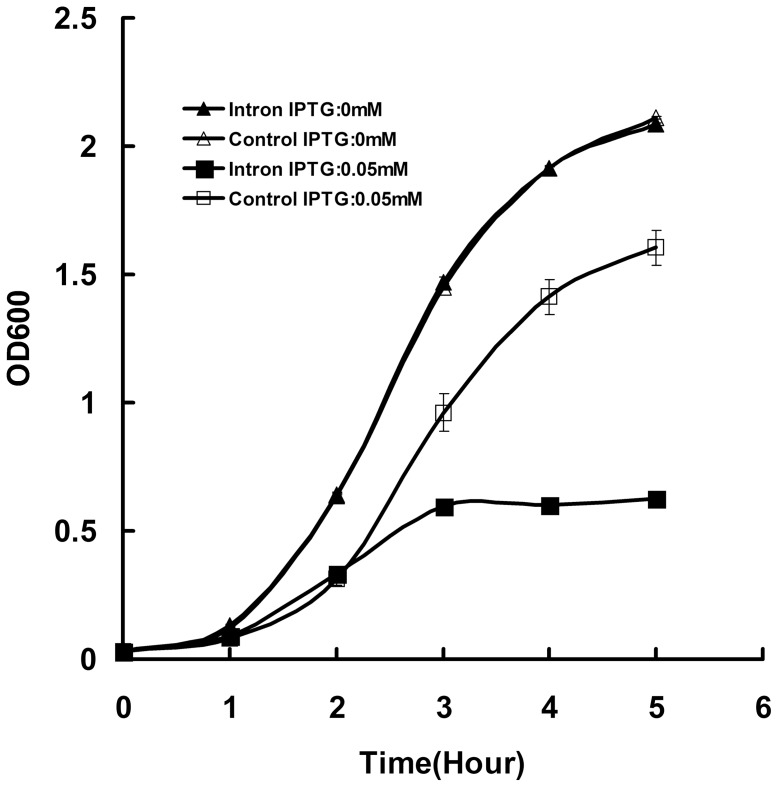
Effect of Pf-WMP3 intron on *E. coli* growth. *E. coli* cells expressing cloned partial Pf-WMP3 *DNAP* gene with intron or an irrelevant control RNA (pET*P4DNAP*) were induced with (0.05 mM) or without IPTG and assayed spectrophotometrically for growth at 37°C over 5 h. *E. coli* growth assays were performed three times and the averaged optical density was used to construct the growth curve. When the error bar cannot be seen, the deviation is less than the size of the symbol.

Like some introns such as 26S rRNA intron from *Tetrahymena thermophila* [25] and 23S rRNA introns from *Coxiella burnetii* [26], the Pf-WMP3 intron displayed a significantly decreased growth rate relative to controls when expressed in *E. coli*. We monitored the growth rates of *E. coli* strains transformed with pET*P3DNAP[+int]* and pET*P4DNAP* spectrophotometrically for 5 h. As shown in Figure 3, *E. coli* expressing the intron had a significant retarded growth rate when compared to the control after 0.05 mM IPTG was added.

### The Pf-WMP3 Intron Encodes a Nicking DNA Endonuclease

**Figure 4:**
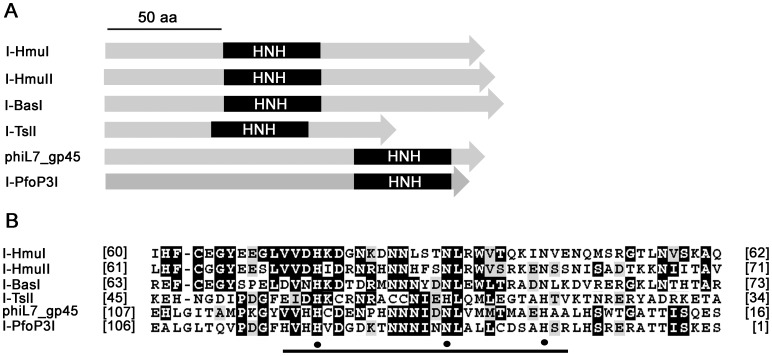
Sequence features of HEases inserted into phage *DNAP* gene introns. A. Shown is I-PfoP3I and highly similar HNH HEases encoded within mobile introns found in *DNAP* host genes from phages SPO1 (I-HmuI), SP82 (I-HmuII), Bastile (I-BasI), W31 (I-TslI) and phiL7 (phiL7_gp45). Black boxes indicate the positions of the HNH motif in these endonucleases. B. Amino acid sequence alignment of the conserved HNH motif. Brackets indicate number of residues in front of and following the aligned sequence. The black bar indicates residues of the active site domain and black spheres indicate the most conserved Asn and His residues. Alignment was generated with Clustalx1.83. Conserved residues are shaded using the BOXSHADE 3.21 program (http://www.ch.embnet.org/software/BOX_form.html). GenBank accession numbers: I-HmuI (YP_002300418.1), I-HmuII (AAA56884.1), I-BasI (AAO93095.1), I-TslI (AAV53690), phiL7_gp45 (ACE75785.1), I-PfoP3I (YP_001285778.1).

As shown in Figure 1, the Pf-WMP3 *DNAP* gene was interrupted by a 672-bp intron, located between Pro363 and Asn364. This intron contains an ORF encoding a 161-amino-acid-residue putative HEase of the HNH family using the protein blast tool at NCBI [27] with default parameters. According to the suggested nomenclature for HEase [28], we named the ORF I-PfoP3I (*I*ntron-encoded HEase, *P*. *fo*veolarum phage Pf-WM*P3*, *I*). I-PfoP3I is inserted into the stem of P6a, which is the same location where the T7-like phages ΦI and W31 encode HEases. There are eight subsets of proteins containing the HNHc domain. Subset 2 has mostly phage proteins which are intron-encoded site-specific endonucleases with the HNHc domain closer to the N-terminal end of the protein in contrast to the other subsets [29]. Figure 4A, B show the conserved HNH motif of I-PfoP3I and other five subset 2 HEases. The intron containing I-PfoP3I, I-HmuI, I-HmuII, I-BasI is inserted in exactly the same genomic position of the respective *DNAP* gene, but from very widely divergent phages.

**Figure 5:**
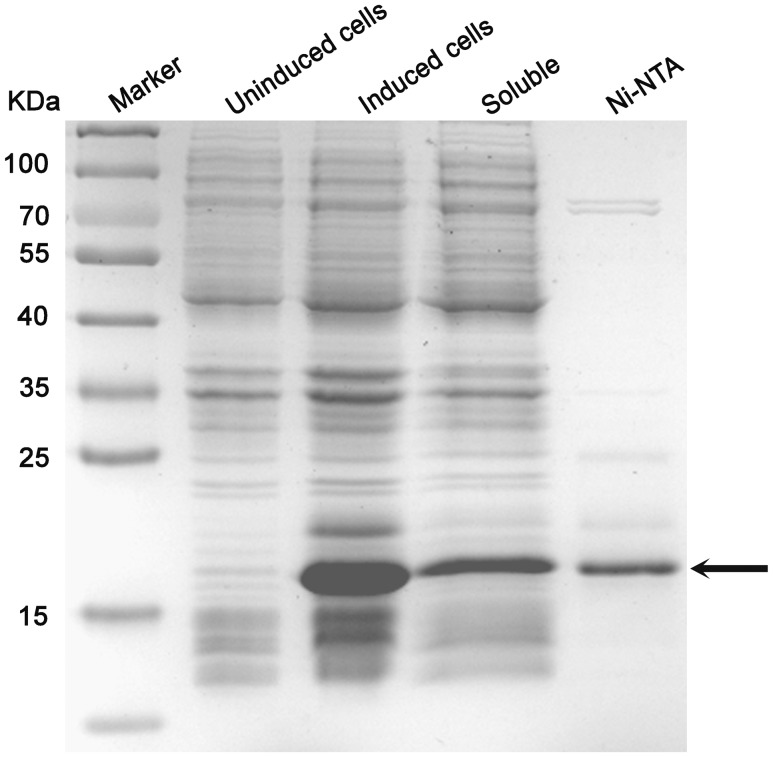
SDS-PAGE of fractions recovered during the purification of His_6_I-PfoP3I. Marker, prestained protein ladder (Thermo); Uninduced, lysate of uninduced cells; Induced, lysate of cells induced with IPTG; Soluble, soluble fraction of the lysate; Ni-NTA, fraction eluted from the Ni-NTA resin. Electrophoresis was performed on a vertical 15% polyacrylamide gel, which was stained with Coomassie Brilliant Blue R-250. The arrowhead denotes the position of the His_6_I-PfoP3I protein.

To address the question of whether the Pf-WMP3 intron ORF encodes a functional endonuclease, I-PfoP3I was expressed including a His_6_ affinity tag at the C-terminal end. The expressed protein product was consistent with its predicted size (18.5 kDa) (Figure 5).

**Figure 6:**
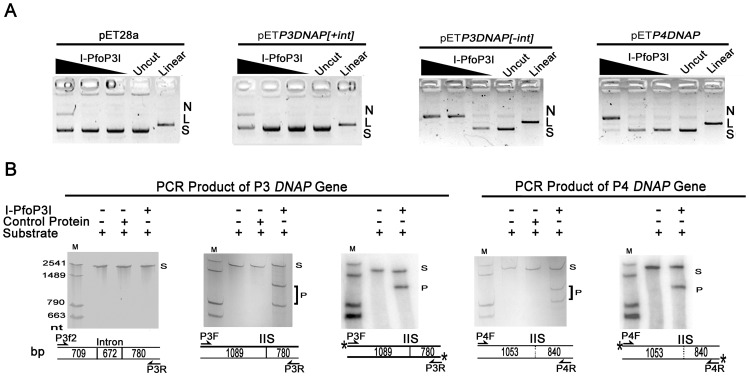
Nuclease activity of I-PfoP3I. A. Cleavage of three kinds of plasmid substrates (10 nM) of intron-plus and intron-minus versions of *DNAP* gene of Pf-WMP3 and the wild-type *DNAP* gene of Pf-WMP4 by I-PfoP3I at 200, 20 and 2 nM. N, nicked; L, linear; S, supercoiled. Uncut plasmids (Uncut) and BamHI-linearized (Linear) plasmids are used as controls at the right. Ethidium bromide-stained agarose gels (1% agarose [wt vol^−1^]) are shown. B. Cleavage of three kinds of PCR product substrates (10 nM) of intron-plus and intron-minus versions of *DNAP* gene of Pf-WMP3 and the wild-type *DNAP* gene of Pf-WMP4 by I-PfoP3I at 1000 nM. The 5′ ends of the ^32^P-labeled strands are marked with an asterisk. Control protein, protein derived from cells transformed with the expression plasmid pET28a(+) vector without insert; M, marker; S, substrate; P, product. The cleavage products were analyzed by PAGE in 6% gel in the presence of 8 M urea. Gels were silver stained or autoradiographed. Schemes of the corresponding DNA substrates are shown on the bottom. IISs are marked with a real line (Pf-WMP3 *DNAP* gene) or a dashed line (homologous site in Pf-WMP4 *DNAP* gene).

Plasmids pET*P3DNAP[+int]*, pET*P3DNAP[−int]* and pET*P4DNAP* were used as substrates to detect supercoiled plasmid DNA cleavage by I-PfoP3I. pET*P4DNAP* contained *DNAP* gene from Pf-WMP4, which was isolated from Lake Weiming as Pf-WMP3 [17]. Both of the two phages infect the freshwater cyanobacterium *P. foveolarum* and they are closely related at the protein level and genome architecture [18]. However, *DNAP* gene from Pf-WMP4 did not contain any IVS. As shown in Figure 6A, a small amount of nicked products of plasmids pET28a and pET*P3DNAP[+int]* were generated by I-PfoP3I at 200 mM after 20 min. I-PfoP3I nicked the intron-minus Pf-WMP3 *DNAP* gene more efficiently than Pf-WMP4 *DNAP* gene. The supercoiled form of plasmid pET*P3DNAP[−int]* was completely converted to other forms by I-PfoP3I at 20 mM after 20 min while pET*P4DNAP* was completely converted to other forms by I-PfoP3I at 200 mM after 20 min.

As shown in Figure 6B, PCR products of Pf-WMP3 *DNAP* gene (intron-plus or intron-minus) and wild type Pf-WMP4 *DNAP* gene were used as substrates (10 nM). One strand of both intron-minus Pf-WMP3 *DNAP* gene and wild type Pf-WMP4 *DNAP* gene were cleaved by purified I-PfoP3I at 1000 nM after 20 min. No cleavage activity was detected on intron-plus Pf-WMP3 *DNAP* gene when incubated with I-PfoP3I under the same condition. No activity was detected using purified protein derived from cells transformed with the expression plasmid pET28a(+) vector without insert (Figure 6B, Lane Control Protein). Substrates with 5′ end-labeled on both strands showed a cleavage product about 1089 nt or 1053 nt in size, indicating that I-PfoP3I introduced a nick in the sense strand of EXON 1 of the target DNA. No cleavage of the antisense strand was detected under the same condition [30].

**Figure 7:**
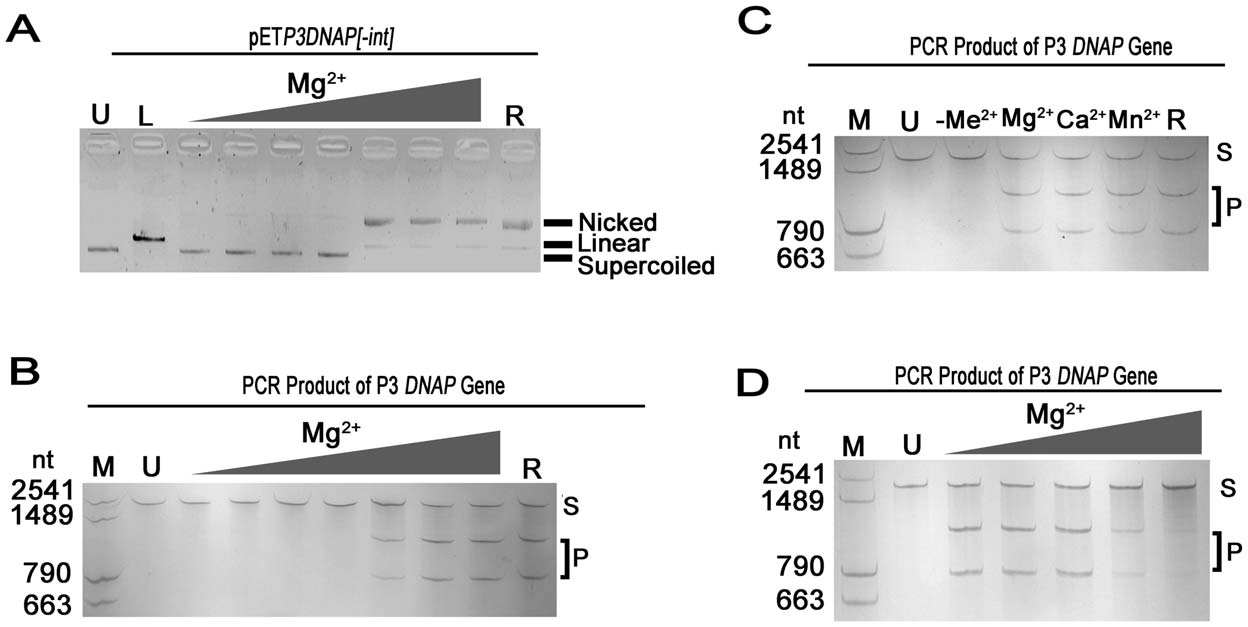
Metal-dependent activity of I-PfoP3I. Plasmid DNA of pET*P3DNAP[−int]* (A) and PCR product of intron-minus version of Pf-WMP3 *DNAP* gene (B, C, D) were used as substrates. U, uncut substrates; L, BamHI-linearized plasmids; R (reference), substrates DNA digested by the metal-containing I-PfoP3I (without EDTA treated) with no externally added divalent ion; M, marker; S, substrate; P, product. Reactions were carried out at a fixed concentration (10 nM) of DNA substrates and (1000 nM) of apo-I-PfoP3I and metal-containing I-PfoP3I. (A, B) The concentrations of Mg^2+^ were 0, 1 pM, 1 nM, 1 µM, 1 mM, 10 mM and 50 mM. C. The effect of various metal ions on the nuclease activity of apo-I-PfoP3I. The reactions were carried out at a fixed concentration (10 mM) of different metal ions Mg^2+^, Ca^2+^, Mn^2+^ or without any metal ion (−Me^2+^). D. Different concentrations of Mg^2+^ (0, 50, 75, 100, 125 mM) were added to metal-containing I-PfoP3I. Lower concentration of Mg^2+^ had no effect on the nuclease activity of I-PfoP3I but over 100 mM Mg^2+^ of inhibited the enzyme activity. Ethidium bromide-stained agarose gels (1% agarose [wt vol^−1^]) (A) or silver stained 6% urea acrylamide gels are shown (B, C, D).

To characterize metal ions effect on the endonuclease activity of apo-I-PfoP3I, purified I-PfoP3I was first treated with EDTA to remove the endogenous ions bound to the enzyme expressed in *E. coli*. ∼1 µM of EDTA remained in the protein solution, extracting any residual metal ions to eliminate any metal contamination. We found that the apo-I-PfoP3I did not cleave plasmid or PCR product DNA (Figure 7A, B, C). However, the endonuclease activity of apo-I-PfoP3I resumed by the presence of one of the metal ions, Mg^2+^, Ca^2+^ or Mn^2+^. The lowest Mg^2+^ concentration used to digest DNA completely is 1000 fold higher than the residual concentration of EDTA. Ca^2+^ and Mn^2+^ were able to activate apo-I-PfoP3I at 10 mM. Co^2+^ and Zn^2+^ make the assay system precipitate (data not shown). To test if the exogenous metal ions would enhance or inhibit endonuclease activity, the enzyme untreated with EDTA was incubated with Mg^2+^ at a concentration range of 0–125 mM. Cleavage analyses indicate that metal-bounding I-PfoP3I expressed in *E. coli* was independent of any divalent cations for activity. Lower concentration of Mg^2+^ had no effect on the nuclease activity of I-PfoP3I, while a higher concentration of Mg^2+^ was progressively detrimental to the enzyme activity. When the Mg^2+^ concentration reached 125 mM, that is ∼10^5^-fold to I-PfoP3I, the Mg^2+^ ion completely inhibited the endonuclease activity (Figure 7D). The metal-bounding enzyme was precipitated in the presence of Mn^2+^, Zn^2+^ or Ca^2+^ (data not shown).

### Mapping of DNA Cleavage Site Introduced by I-PfoP3I

**Figure 8:**
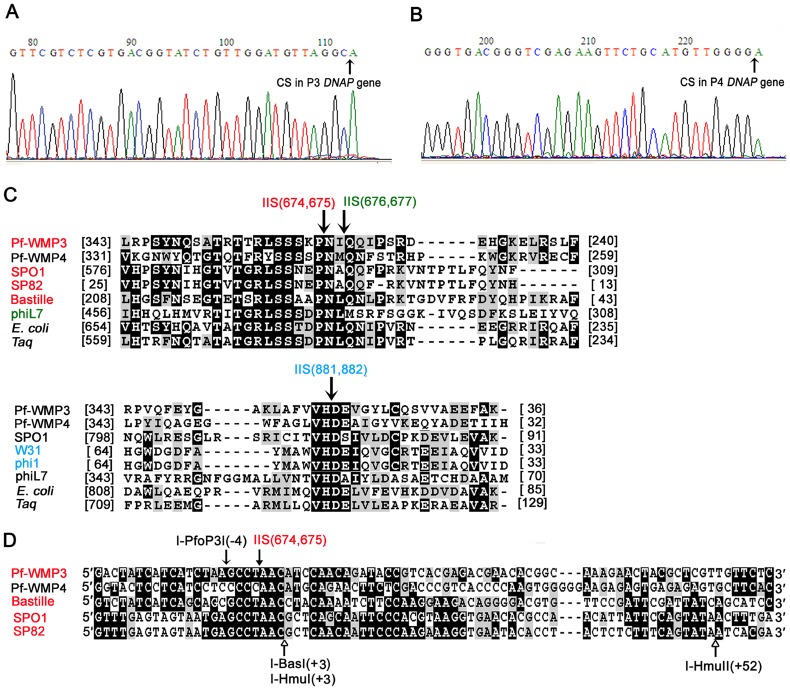
The cleavage sites and the host target site reading frame. (A, B) Determination of the cleavage sites by direct DNA sequencing of cleaved PCR products of Pf-WMPP3 *DNAP* gene and Pf-WMP4 *DNAP* gene using reverse primers P3r1 and P4r1, respectively. *Taq* DNA polymerase was used for sequencing and a non-template 3′ A was added. C. The corresponding amino acid sequence alignment for related *DNAP* genes, including the three IISs. The alignment was generated and the conserved residues were shaded as before. GenBank accession numbers: Pf-WMP3 (ABQ12452), Pf-WMP4 (YP_762649), SPO1 (P30314), SP82 (S53691), Bastille (AAO93094.1), phiL7 (YP_002922660), *E. coli* (P00582), *Taq* (BAA06775). The arrows indicate the IIS. Numbers in parenthesis indicate *E. coli* numbering. Organisms and their IISs in the *DNAP* gene are in the same color. Numbers in brackets indicate numbers of residues in front of and following the aligned sequence. D. Comparison of DNA cleavage sites of four HNH endonucleases encoded in phage group I introns in *DNAP* genes. The sequences of the coding strands of five *DNAP* genes are presented. The alignment was generated and the conserved residues were shaded as before. GenBank accession numbers for nt sequences as follows: Pf-WMP3 (EF537008.1: 9191.10279, EF537008.1: 10952.11731), Pf-WMP4 (NC_008367.1: 10336.12228), Bastille (AY256517), SPO1 (SP1GP31A), SP82 (BSU04812). The filled arrows indicate IIS, the open arrows indicate cleavage position of the Pf-WMP3 intron endonuclease (I-PfoP3I) and the open arrowheads indicate the SPO1 intron endonuclease (I-HmuI), the Bastille intron endonuclease (I-BasI) and the SP82 intron endonuclease (I-HmuII). Only I-PfoP3I nicks the coding strand in EXON 1 while the other three HNH HEases nick the template strand in EXON 2.

As shown in endonuclease activity assays, the breakpoints introduced by I-PfoP3I were located on the coding strands of both Pf-WMP3 and Pf-WMP4 *DNAP* genes. Nucleotide sequencing was used to determine precise cleavage sites of I-PfoP3I. Both substrates were cleaved on the coding strands 4 nt upstream of the IIS despite considerable differences in the nucleotide sequence surrounding the cleavage site (Figure 8A, B). Pf-WMP3 intron was inserted in the same site as introns in SPO1, SP82 and Bastille according to the corresponding amino acid sequence alignment for related genes (Figure 8C). The fact that both substrates were cleaved at the same site of both Pf-WMP3 and Pf-WMP4 *DNAP* genes indicates that I-PfoP3I binds homologous stretches of its respective *DNAP* genes.

**Figure 9:**
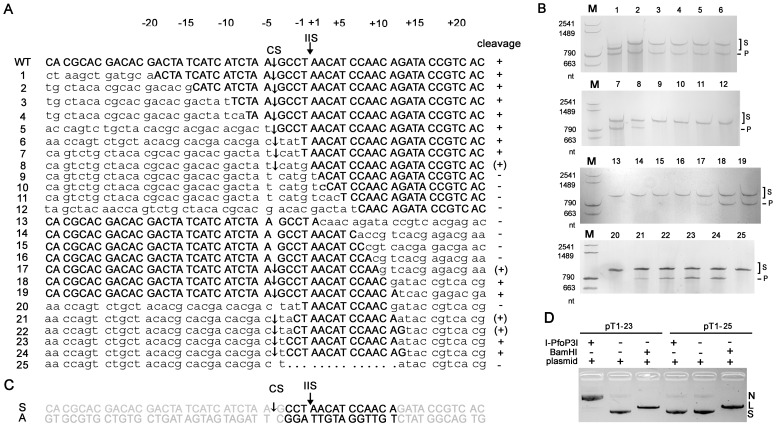
Mapping of the minimal sequence required for I-PfoP3I cleavage. A. Recognition site sequences. The intron-less sequence of the sense strand of the Pf-WMP3 *DNAP* gene is shown. Wild-type (WT) sequence is indicated in upper case. The portion of each sequence that is out of register as a result of deletion is indicated in lower case. The filled arrow indicates IIS. The open arrows indicate cleavage site (CS) of I-PfoP3I. Cleavage is indicated with + for efficient cleavage, (+) for low or nearly no cleavage and – for no detected cleavage. B. Polyacrylamide gels showing products from cleavage assays with short wild-type flanking sequences either upstream or downstream of the IIS. The substrate lengths are successively shortened by 1 to 81 base pairs for each substrate (S) tested as specified in A. Note that only one cleavage product (P) was visualized, as the other strand was less than 180 nt in size and ran out of the gel by electrophoresis. I-PfoP3I cleavage sites on substrates (5, 6, 7) were determined by direct DNA sequencing using reverse primers P3r1 (Figure S2). C. Summary of recognition site mapping. S, sense strand; A, antisense strand. The region of the target site is indicated in bold. The filled arrows indicate IIS. The open arrows indicate cleavage site (CS) of I-PfoP3I. D. Cleavage of plasmid substrates (10 nM) of pT1-23 and pT1-25 by I-PfoP3I at 20 nM. N, nicked; L, linear; S, supercoiled. An ethidium bromide-stained agarose gel (1.5% agarose [wt vol^−1^]) is shown.

To map the approximate size of the recognition site, we focused our investigation on a 30 bp region surrounding the I-PfoP3I cleavage site in intronless Pf-WMP3 *DNAP* gene. We used a PCR-based mutagenesis strategy to introduce site-directed 1 to 81 bp deletions into the region to make short wild-type flanking sequences either upstream or downstream of the IIS (Figure 9A). We presumed that deletion of base pairs within the recognition site would greatly reduce or eliminate cleavage of the substrate by I-PfoP3I [14]. Figure 9B shows that PCR products containing deletions extending from positions −3 to +11 (with respect to IIS) were cleaved with reduced efficiency (Figure 9B 8, 17, 21 and 22) or not cleaved by I-PfoP3I. Targets with deletions outside this region including base pairs around the cleavage site (Figure 9B 5–7, 23 and 24) showed full activity of I-PfoP3I cleavage. The results indicate that the sequence necessary for full cleavage activity by I-PfoP3I comprises at least 14 bp (Figure 9C). As shown in Figure 9D, plasmids pT1-23 and pT1-25 were used as substrates to detect supercoiled plasmid DNA cleavage by I-PfoP3I. pT1-23 contained the minimal recognition sequence and pT1-25 contained the same sequence with 14 bp sequence deleted (Figure 9A). I-PfoP3I nicked pT1-23 efficiently, confirming the short nicking site.

### Phylogenetic Relationships of *DNAP* Gene Group I Introns and their HNH Proteins

**Figure 10:**
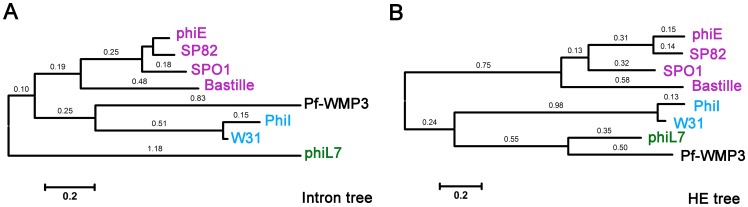
Phylogenetic relationships of group I introns inserted into phage *DNAP* genes (A) and their HNH proteins (B). All introns belong to IA2 subgoup of group I introns. The trees include only those HNH HEG-containing group I introns that are inserted in *Bacillus* phages (purple), *Enterobacteria* phages (blue), *Xanthomonas* phage (green) and cyanophage Pf-WMP3 (black). Horizontal distances are proportional to evolutionary divergence expressed as substitutions per site. Branch lengths higher than 0.1 are indicated on the tree branches. The scale bars represent 0.2 fixed mutations per amino acid position. GenBank accession numbers for intron sequences as follows: phiE (U04813.1), SP82 (BSU04812), SPO1 (M37686:), Bastille (AY256517), Pf-WMP3 (EF537008.1), phiI (AY769989.1), W31 (AY769990.1), phiL7 (EU717894.1). GenBank accession numbers for HEase sequences as follows: phiE (AAA56886.1), phiI (AAV53690.1), W31 (AAV53693.1). A phylogenetic tree was constructed using the neighbor-joining method in MEGA 4.

The trees (Figure 10) demonstrated that most introns and HNH proteins in *DNAP* gene of phages infecting the same host appeared to be more closely related. However, HNH proteins were closely related in Pf-WMP3 and phiL7, which are biogeographically and morphologically distantly related. Cyanophage Pf-WMP3 of *Podoviridae* family, infecting the freshwater cyanobacterium *P. foveolarum*, was found in Beijing, China. The lytic phage phiL7 of *Siphoviridae* family, infecting *Xanthomonas campestris* pv. campestris, was isolated in the laboratory in Taiwan [6]. The introns in Pf-WMP3 and PhiL7 are inserted in a similar position of their respective *DNAP* genes and the HEases have a similar location of the HNH motif in the C-terminal half. It is possible that their HNH motifs and their DNA recognition sequences may be related.

## Discussion

### A group I Intron in the Genome of Cyanophage Pf-WMP3

Self-splicing group I introns are rarexly found in T7-like phages. In this study, we show that the *DNAP* gene, an essential enzyme for the replication for phage DNA, carries a group I intron that is efficiently spliced *in vivo* and *in vitro*. The intron is inserted in the *DNAP* gene at the site 674 (*E. coli* numbering), homologous to the introns in SPO1, SP82 and Bastille, all of which are closely related phages infecting *B. subtilis.* Other two group I introns inserted in *DNAP* genes of ΦI and W31 are at the site 881 [7]. The group I intron in *Xanthomonas campestris* phage phiL7 is inserted at the site 676 [6], indicating that there are at least three sites within these genes that can contain intron insertions (Figure 8C). Like other group I introns [31], [32], the insertion site is located in a highly conserved region of functional importance within the coding sequence. According to the crystal structure of a bacteriophage T7 DNA replication complex, the insertion site is in the finger subdomain of DNAP [33]. It is worth noting that the first three nucleotides of the intron are UAA, which can also serve as the stop codon of EXON 1 if this intron does not splice efficiently due to inexact deletion. This might be lethal, for EXON 1 displayed DNA exonuclease activity without any synthesis activity *in vitro* (data not shown).

There are some hypotheses to explain the growth inhibition caused by Pf-WMP3 intron. For example, the HEase activity from I-PfoP3I, with its relatively short recognition sequence, might cleave essential *E. coli* genes (in particular the DNA *PolI* gene would be a likely candidate). Also, when proteins are hyperexpressed from plasmids, a common observation is cessation of cell growth, even when the proteins are not toxic (beta-galactosidase, for example). Further work attempting to test the effect of group I introns on changes on the fitness of the host organism would be performed.

### A Functional HEase Encoded by this Intron

A BLASTP search of the protein database using the I-PfoP3I amino acid sequence revealed only one protein to be highly similar, with maximum similarity in the C-termini, which contain the HNH motif, but conservation extending throughout, extending through the N-termini (which presumably contain the DNA binding regions). Interestingly, this presumptive HEase is from phage phiL7, whose intron is inserted into the homologous region of its *DNAP* gene. The E values of the subsequent BLASTP hits are much lower. The next two have good alignments with the HNH at their C-termini. But the very next one which is from *Natromonas* aligns its N-terminal HNH region with the C-terminal motif in I-PfoP3I, as do almost all the remaining hits on the list. The proteins in this list are referred to as “putative” HEases or “hypothetical” proteins because their biochemical activities have not been determined. The co-crystal structure of I-HmuI, which includes the HNH motif in the N-terminal part (Figure 4A), displays a 2-domain arrangement with N-terminal catalytic and C-terminal DNA-binding domains (although significant specific DNA contacts are made near the N-terminus), leading to the proposal that these phage endonucleases have a two-domain structure [34].

Apo-I-PfoP3I required one of the metal ions, Mg^2+^, Ca^2+^ or Mn^2+^ for endonuclease activity, indicative of a relatively relaxed divalent metal requirement (Figure 7C). However, the fact that I-PfoP3I expressed and purified from *E. coli* cleaved DNA substrates independently of any divalent cations indicates that endogenous metal is sufficient for promoting the activity. A higher concentration of Mg^2+^ is progressively detrimental to the enzyme activity (Figure 7D).

From the results presented in Figure 6, we suggest that I-PfoP3I possess nicking activity *in vitro* as DNA endonucleases I-HmuI, I-HmuII and I-BasI encoded in the introns of phages SPO1, SP82 and Bastille respectively. All these five HEases belong to the HNH endonuclease family [2], [35]. I-PfoP3I was unable to cleave intron-plus DNA, indicating the disruption to the target site caused by the acquisition of the intron. Although the plasmid pET*P3DNAP[+int]* became relaxed circular after exposure to the enzyme (Figure 6A), the nick could have occurred anywhere in the plasmid, not necessarily at the normal cleavage site of the enzyme. The recognition sequence for this enzyme is short (Figure 9) and a secondary cleavage site could be recognized at very high enzyme concentrations. I-HmuI, I-HmuII and I-BasI cleave the template strand of the homologous alleles [30], [34]. I-PfoP3I produced a nick in the coding strand like HNH HEase I-TwoI which is encoded in *nrdE* gene of *Staphylococcus aureus* phage Twort [31]. The incision that each of the five HNH HEases generates is 5′ of the IIS, independent of which stand is cleaved [31]. Cleavage of the recipient DNA with HEase encoded within the intron makes the intron spread to cognate intron-less genes and persists in a host gene. As shown in Figure 8D, we compared the nt sequence flanking the cleavage site of the Pf-WMP3 and Pf-WMP4 *DNAP* genes to those of HEases residing within group I introns of *DNAP* genes such as I-HmuI, I-HmuII and I-BasI. I-HmuI cleaves 3 nt downstream of the IIS on both SPO1 and SP82 *DNAP* genes in a region with few differences between them [11]. In contrast, I-PfoP3I cleaves 4 nt upstream of the IIS on both intron-minus Pf-WMP3 *DNAP* gene and Pf-WMP4 *DNAP* gene in a region with more differences between these two *DNAP* genes, indicating the tolerance of multiple substitutions within the target sequences. From the results in Figure 9, we suggest that the recognition site of I-PfoP3I covers 2 to 15 bps downstream of the cut site (i.e. the recognition site covers 3 bp upstream and 11 bp downstream of IIS).

### The Intron-HEG Element Might Insert in Pf-WMP3 Genome Individually after Differentiation from Pf-WMP4

Phylogenetic analysis suggests that these intron-HEG elements have been transferred horizontally among phages infecting similar hosts, indicating these elements can continue to persist into new populations or species via horizontal transfer [36]. Pf-WMP4 is closely related to Pf-WMP3 in genome sequence, size and structure. However, Pf-WMP4 genome does not contain any IVS, suggesting that the intron-HEG element in Pf-WMP3 genome might be obtained after differentiation of these phages. Although Pf-WMP3 and *Xanthomonas campestris* phage phiL7 contain close IISs and closely related HNH proteins, the group I introns of these two phages are distantly related (Figure 10). This might favor the model that the chimeric mobile element was formed by group I introns and HEGs individually targeting the same set of highly conserved DNA sequences for insertion and cleavage respectively [37].

In cyanophages, HEases were only reported as free-standing HEases such as F-CphI found in S-PM2 [4] and some similar HEases. All of these HEases are encoded adjacent to an intron-containing *psbA* gene, encoding the D1 core component of the photosynthetic reaction center PSII (photosystem II) [5]. Apart from these HEases, this is the firsxt report of the presence of a functional HEase encoded in a group I self-splicing intron in a protein-coding gene (*DNAP* gene) in a cyanophage genome.

## Materials and Methods

### Cyanobacterial Strains, Phages, Media and General Methods

*P. foveolarum* was purchased from the Freshwater Algae Culture Collection of the Institute of Hydrobiology, Chinese Academy of Sciences. It was inoculated in BG11 medium supplemented with NaNO_3_ (1.5 g l^−1^) and incubated at 28°C under continuous fluorescent light (28 microeinsteins m^−2^s^−1^) [38]. Pf-WMP3 and Pf-WMP4 were obtained from a surface water sample from Lake Weiming in Peking University of Beijing City in People’s Republic of China, on July 22, 2003. After water samples were filtered through a 0.22- filter (Millipore), the filters were mixed with exponentially growing cultures of *P. foveolarum* and then spread onto BG11 agar plates. Plaque formation was used to isolate phages [39].

### Isolation of Pf-WMP3 and Pf-WMP4 Phage DNA

To obtain a template for PCR reactions, genomic DNA was isolated from Pf-WMP3 and Pf-WMP4 according to previously published methods [40], [41]. Briefly, after the addition of MgSO_4_ (final concentration 20 mM) to lysates, phage particles were precipitated using polyethylene glycol grade 6000 (PEG 6000) and then further purified by sucrose density gradient. Purified phage particles were broken with SDS and proteinase K. DNA was extracted with phenol-chloroform and precipitated with NaOAc and ethanol. The purified DNA was then resuspended in sterile H_2_O and stored at −20°C.

### Plasmid Construction

Intron P3f2 and P3R primer sites flank the intron sequence 709 bp upstream and 780 bp downstream respectively (Figure 1). Pf-WMP3 genomic DNA was used as a template for PCR with a pair of primers P3f2 and P3R. The PCR product was cloned into pEASY-T1 cloning vector (TRANS) to yield plasmid pT*P3DNAP[+int]* and the orientation of cloned *P3DNAP[+int]* gene was confirmed by DNA sequencing. This intron sequence was then subcloned into pET28a(+) vector (Novagen) and the resulting plasmid was termed pET*P3DNAP[+int]*.

The intronless version of Pf-WMP3 *DNAP* gene was amplified using an overlapping extension technique of PCR [42] and the PCR product of Pf-WMP3 *DNAP[−int]* gene was digested with restriction enzymes BamHI and SalI and then was ligated into a pET28a(+) vector to yield plasmid pET*P3DNAP[−int]*. PCR product of wild type Pf-WMP4 *DNAP* gene without any IVS was digested with restriction enzymes BamHI and SalI and then was ligated into a pET28a(+) vector to yield plasmid pET*P4DNAP*.

*I-PfoP3I* gene was obtained from Pf-WMP3 intron using primers P3f4 and P3r3 and the PCR product was digested with restriction enzymes NcoI and XhoI and then cloned into a pET28a(+) vector to generate plasmid pET*I-PfoP3I* which was used for overexpression of I-PfoP3I.

Targets (23, 25) used to determine the I-PfoP3I recognition site boundaries were cloned into pEASY-T1 cloning vector (TRANS) to yield plasmids pT1-23, pT1-25.

### *In vivo* Splicing Assay

*P. foveolarum* was grown in BG11 medium at 28°C to an OD_600_ of 1.0 and infected with Pf-WMP3 at a multiplicity of 8 per cell. RNA was isolated at various times after infection using TRNzol Reagent (TIANGEN). RNase-free DNaseI (TaKaRa) was used to remove contaminating DNA. The total RNA was then incubated with sequence-specific primer P3r1 (Figure 1). Reverse transcription was carried out using M-MLV Reverse Transcriptase (Promega) according to the manufacturer’s recommendations. PCR was used to analyze the presence of spliced and unspliced products. The primers P3f1 and P3r1 were used. *Taq* DNA polymerase was purchased from TIANGEN. The products were analyzed by electrophoresis in a 2% agarose gel and visualized with ethidium bromide.

### *In vitro* Transcription and Splicing Assay

The P3f3 and P3r2 primers flank the intron 184 bp upstream and 128 bp downstream respectively (Figure 1). The pre-RNA for the *in vitro* splicing experiment was prepared by transcription using Riboprobe® *in vitro* Transcription System-T7 (Promega). The XhoI linearized pET*P3DNAP[+int]* (1 µg) was incubated with 40 mM Tris-HCl (pH 7.9), 10 mM NaCl, 6 mM MgCl_2_, 2 mM spermidine, 10 mM DTT, 40 u Recombinant RNasin Ribonuclease Inhibitor, 0.5 mM each of rATP, rGTP, rCTP and rUTP, 20 u T7 RNA Polymerase in a total volume of 20 µl at 37°C for 1 h. Template DNA was digested with RNase-free DNaseI (Promega). Reverse transcription was performed using sequence-specific primer P3r2 and M-MLV Reverse Transcriptase (Promega) according to the manufacturer’s recommendations. PCR was carried out using primers P3f3 and P3r2. The resulting products were analyzed by electrophoresis in a 2% agarose gel.

### Prediction of Intron Secondary Structure

Prediction of the intron secondary structure was performed using Mfold default settings (http://www.bioinfo.rpi.edu/applications/mfold/rna/form1.cgi) and modified by hand using published intron secondary structures as a reference and was drawn using Adobe Photoshop (version 7.0).

### *E. coli* Growth Assay

*E. coli* BL21 (DE3) transformed with pET*P3DNAP[+int]* and pET*P4DNAP* were grown overnight at 37°C in Luria-Bertani broth plus 50 µg ml^−1^ kanamycin. Cells were then used to inoculate 50 ml fresh Luria-Bertani broth with 50 µg ml^−1^ kanamycin and 0.05 mM isopropyl-beta-D-thiogalactopyranoside (IPTG; Merck). The starting OD_600_ (optical density at 600 nm) value was 0.03. Growth of IPTG-induced cells was assayed spectrophotometrically at 600 nm every hour for 5 h (37°C, shaking) [26].

### Expression and Purification of I-PfoP3I

*E. coli* BL21 (DE3) bacteria transformed with pET*I-PfoP3I* were grown in Luria-Bertani broth supplemented with 50 µg ml^−1^ kanamycin at 37°C until the density reached an OD_600_ of 0.6. Expression of His_6_I-PfoP3I was induced by adding IPTG to a final concentration of 0.1 mM. After an additional 4 h of culture growth at 30°C, cells were harvested and disrupted by sonication in lysis buffer (50 mM NaH_2_PO_4_ (PH 8.0), 300 mM NaCl, 10 mM imidazole). The lysate was centrifuged at 10 000 g for 30 min at 4°C to pellet the cellular debris. The soluble fraction was added with Ni-NTA resin (Novagen) and then mixed gently for 30 min at 4°C. The resin was settled by low speed centrifugation (1000 g) for 10 s and then was washed several times with wash buffer (50 mM NaH_2_PO_4_ (PH 8.0), 300 mM NaCl, 20 mM imidazole). The protein was eluted with elution buffer containing high concentrations of imidazole (50 mM NaH_2_PO_4_ (PH 8.0), 300 mM NaCl, 250 mM imidazole). Protein concentration was determined using the Bradford method [43].

### EDTA Treatment of the Purified I-PfoP3I

To examine the effect of a single divalent metal ion on the enzymatic activity of I-PfoP3I, preparation of apo-enzyme without any metal ion cofactor is necessary. The concentrated I-PfoP3I (∼0.6 mg ml^−1^) was incubated with 1 M divalent metal chelating agent EDTA at room temperature for 1 h. The EDTA-treated I-PfoP3I was dialyzed against 1 l of 10 mM Tris-HCl buffer (pH 8.0) three times at 4°C overnight. A residual EDTA concentration of ∼1 µM remained in the protein solution to ensure the absence of contamination of divalent metal ions. After dialysis, the apo-enzyme sample was concentrated to 1 ml (0.3 mg ml^−1^) by an Ultrafree-15 centrifugal filter (Millipore, Bedford, MA, USA).

### Endonuclease Assays

For endonuclease assays, 10 nM plasmids pET28a, pET*P3DNAP[+int]*, pET*P3DNAP[−int]*, pET*P4DNAP* and were used as substrates. Cleavage reactions were carried out in 50 mM Tris-Cl pH 8.0, 5 mM DTT at 200, 20 and 2 nM enzyme and incubated at room temperature for 20 min. Reactions were stopped by the addition of 5 µl of proteinase K (20 mg ml^−1^) and further incubated at 37°C for 1 h. BamHI digested pET28a, pET*P3DNAP[+int]*, pET*P3DNAP[−int]*, pET*P4DNAP* were used as controls. After ethanol precipitation, samples were analyzed by electrophoresis in a 1% agarose gels [wt vol^−1^] stained with ethidium bromide and analyzed with ImageJ 1.42q.

The double-stranded DNA endonuclease activity of I-PfoP3I was assayed using purified PCR product with or without intron sequence of Pf-WMP3 and Pf-WMP4 *DNAP* genes. Both of the two strands were labeled with [γ-^32^P] ATP at 5′ end. I-PfoP3I was incubated with 4000 counts per minute (cpm) of the fragments in 50 µl of assay buffer (50 mM Tris-Cl pH 8.0, 5 mM DTT) at room temperature for 20 min. Reactions were stopped by the addition of 5 µl of proteinase K (20 mg ml^−1^) and further incubated at 37°C for 1 h. After ethanol precipitation, samples (1∶1 added with TaKaRa RNA Loading Buffer) were loaded on a 6% acrylamide gel with 8 M urea and separated in 40 mM Tris-borate pH 8.0, 2 mM EDTA. Products were visualized with silver staining or autoradiography. Although the use of silver staining to visualize DNA makes images with higher background, this staining method is a rapid (less than 30 min to visualize DNA after urea acrylamide gel electrophoresis), sensitive, reproducible and inexpensive alternative to radioactive, fluorescent and chemiluminescent detection approaches.

To exactly localize the cleavage sites introduced by I-PfoP3I, double-stranded DNA endonuclease activity products of intron-minus Pf-WMP3 *DNAP* gene and Pf-WMP4 *DNAP* gene were sequenced directly using ABI *3730xl* DNA Analyzer. Reverse primers P3r1 and P4r1 were used as sequencing primers respectively.

### Characterization of the Effects of Divalent Metal Ions on Endonuclease Activity

Five divalent metal ions (Co^2+^, Mg^2+^, Mn^2+^, Ca^2+^, Zn^2+^) were used for these tests. The activity assays were carried out with apo-I-PfoP3I and different kinds of metal ions respectively. Reactions without any divalent metal ions were used as controls.

### Mapping of the Recognition Site

Targets used to determine the I-PfoP3I recognition site boundaries contained variants of the wild-type intronless sequence differing by 1 to 81 bp deleted from the putative recognition site. The upstream parts of the deleted site were amplified using universal forward primer P3f3 and primers 1r to 25r. The downstream parts of the deleted site were amplified using primers 1f to 25f and universal reverse primer P3R. Oligonucleotides are shown in Table S1. The final targets containing altered target sites with deletions between positions −19 and +11 (with respect to the IIS) were created by amplification of the downstream parts and the upstream parts using forward primer P3f3 and reverse primer P3R. Reactions were cycled 35 times at 94°C for 30 s, 58°C for 30 s and 72°C for 60 s with a final extension at 72°C for 10 min. Amplification products were confirmed by sequencing (for variant target site sequences, see Figure 9A). Cleavage reactions were performed as above. Reaction products were separated by gel electrophoresis on a 6% acrylamide gel with 8 M urea and separated in 40 mM Tris-borate pH 8.0, 2 mM EDTA. Products were visualized with silver staining. Note that only one cleavage product was visualized, as the other strand was less than 180 nt in size and ran out of the gel by electrophoresis. Substrates (10 nM) of plasmids pT1-23 and pT1-25 were cleaved by I-PfoP3I at 20 nM. Cleavage reactions were performed as above. BamHI digested pT1-23 and pT1-25 were used as controls. Samples were analyzed by electrophoresis in a 1.5% agarose gels [wt vol^−1^] stained with ethidium bromide.

### Oligonucleotides

The following oligonucleotides were used:

P3F, 5′-TGGGGGATCCATGAACATCTTCGGGCATACT;

P3f1, 5′- ACGCACGACACGACTATCATCATCTAAGCC;

P3f2, 5′-AAGGGGTACCTGAGGTGTAT CGTCCTGATC;

P3f3, 5′-TACGTCATCAGAGGTACTGGAAGAA;

P3f4, 5′- AGGGCCATGGGAATGGACGATAAACTGAAGGA;

P3R, 5′- TTTCGTCGACCTACTTCGCCTCTAGCCATGT;

P3r1, 5′- ATCGTAGTACATCTCCATGTACGCTGCCAT;

P3r2, 5′- TACGCTGCCATGATACGTAGCTGGA;

P3r3, 5′- TTCCCTCGAGGACCCTACTCTCTTTCGAGATA;

P4F, 5′- CGGATCCATGACTACTTACATCGCGCT;

P4R, 5′- CGTCGACTTCATTTGTCGCTCCAGGAA;

P4r1, 5′- GAACTCATCAGGGTTGCTCTCACGA.

## Supporting Information

Figure S1 **Total nucleotide sequence of the Pf-WMP3** ***DNAP*** **gene.** Predicted amino acid sequences are given below. Stop codons are indicated with “End”. Intron 5′ and 3′ splice sites are indicated with filled arrows. The line under AGGAGGT indicates the putative ribosomal binding site (RBS). GenBank accession no. EF537008.1: 9191.11731.
(DOC)

Figure S2 **Determination of the cleavage sites (CS) by direct DNA sequencing of cleaved PCR products of Pf-WMP3** ***DNAP*** **gene with deletions (** **Figure 9** **, Line 5, 6, 7) using reverse primers P3r1.** The open arrows indicate cleavage position of I-PfoP3I.
(TIF)

Table S1 **Oligonucleotides used to determine the I-PfoP3I recognition site boundaries contain variants of the wild-type intronless sequence.**
(DOC)
