# Supplementary material for: I-PfoP3I: A Novel Nicking HNH Homing Endonuclease Encoded in the Group I Intron of the DNA Polymerase Gene in Phormidium foveolarum Phage Pf-WMP3
Source: PLoS One. 2012 Aug 27;7(8):e43738. doi: 10.1371/journal.pone.0043738 (PMC3428280; doi:10.1371/journal.pone.0043738)
Supplement: Figure S1 — Total nucleotide sequence of the Pf-WMP3 DNAP gene. Predicted amino acid sequences are given below. Stop codons are indicated with “End”. Intron 5′ and 3′ splice sites are indicated with filled arrows. The line under AGGAGGT indicates the putative ribosomal binding site (RBS). GenBank accession no. EF537008.1: 9191.11731. (DOC) [file pone.0043738.s001.doc]

1 ATG AAC ATC TTC GGG CAT ACT GTA CCT GTA GTA CTC GAC ATC GAA 45

1 Met Asn Ile Phe Gly His Thr Val Pro Val Val Leu Asp Ile Glu 15

46 AGT GAT GAC CTA TCA GCA CAG TAC GAT GTA GAT ACA CGG GTC TAC 90

16 Ser Asp Asp Leu Ser Ala Gln Tyr Asp Val Asp Thr Arg Val Tyr 30

91 CTG ATA GGT GTC CAG TTC CTA GCA GAC TAC GGA CTA TAT GCA GCA 135

31 Leu Ile Gly Val Gln Phe Leu Ala Asp Tyr Gly Leu Tyr Ala Ala 45

136 GGT GAC TAT GTT TAC GGT AGC TTA GAG GAG ATG CGT GAC CTT TGT 180

46 Gly Asp Tyr Val Tyr Gly Ser Leu Glu Glu Met Arg Asp Leu Cys 60

181 GTG TCT CTC GCT AAC GAC ACA GAT GTA ACG TTC GTT ATC CAT AAC 225

61 Val Ser Leu Ala Asn Asp Thr Asp Val Thr Phe Val Ile His Asn 75

226 GCG TCG TTC GAT GTA CCC GCG TTA CGC CTC AGA GGC ATA CCT ATC 270

76 Ala Ser Phe Asp Val Pro Ala Leu Arg Leu Arg Gly Ile Pro Ile 90

271 AAG CGT TAC TTC TGT ACA CAA GTC GCA GCA CAT ACA TGG CAT CCT 315

91 Lys Arg Tyr Phe Cys Thr Gln Val Ala Ala His Thr Trp His Pro 105

316 AAG TCT AGT GAA GAG AAC AGT CTA GAC AGT CTA ACT GGT ATG AAG 360

106 Lys Ser Ser Glu Glu Asn Ser Leu Asp Ser Leu Thr Gly Met Lys 120

361 CTT GAC CTA CGG GCT GAG CTT GAG GTG TAT CGT CCT GAT CTC AAA 405

121 Leu Asp Leu Arg Ala Glu Leu Glu Val Tyr Arg Pro Asp Leu Lys 135

406 GGT AAA CCA AAG GGT TAT GAG TAC AGA CTT TAT GAC GAC AAA AGC 450

136 Gly Lys Pro Lys Gly Tyr Glu Tyr Arg Leu Tyr Asp Asp Lys Ser 150

451 CAT AGG GGT TGG TCA ACG GTT AAC GAT ATC TTC CAT AGT TAT CTT 495

151 His Arg Gly Trp Ser Thr Val Asn Asp Ile Phe His Ser Tyr Leu 165

496 GTG TCT GAT CTT CAA GCT ACA GGT AAG CTC TAC ACT ACG TTG GAG 540

166 Val Ser Asp Leu Gln Ala Thr Gly Lys Leu Tyr Thr Thr Leu Glu 180

541 GAT AAC TTC AGC AGT GAT GAA CGA GCG TTA CGG TGT TTA CTC GAC 585

181 Asp Asn Phe Ser Ser Asp Glu Arg Ala Leu Arg Cys Leu Leu Asp 195

586 ATC AAC CAA CCG TAT GTC GAA CTC ATC ATC GAG TTT GAA CAA GGC 630

196 Ile Asn Gln Pro Tyr Val Glu Leu Ile Ile Glu Phe Glu Gln Gly 210

631 TCA TGG GTT GAT GTT GAC CGA GTA GAT GAA GTA CAG TCT AAG CTA 675

211 Ser Trp Val Asp Val Asp Arg Val Asp Glu Val Gln Ser Lys Leu 225

676 CAG ACA CTA CGT GAT GAA GCG TGG TCT GAG ATA ACA ACA CAC ATA 720

226 Gln Thr Leu Arg Asp Glu Ala Trp Ser Glu Ile Thr Thr His Ile 240

721 CGC TAC GAT GTA ACA GAG ATA AAG CGA TAC AAG AAC GGG TAC AAG 765

241 Arg Tyr Asp Val Thr Glu Ile Lys Arg Tyr Lys Asn Gly Tyr Lys 255

766 AAA CGC AAC GGT GTA GTG ACA TAT GAC CAC TGC CCT CTT GTT GAC 810

256 Lys Arg Asn Gly Val Val Thr Tyr Asp His Cys Pro Leu Val Asp 270

811 TTC AAC CCC GGC AGT AGT GAC CAA GTA GCA GCA GCA CTG ACA TAC 855

271 Phe Asn Pro Gly Ser Ser Asp Gln Val Ala Ala Ala Leu Thr Tyr 285

856 CTG TAT GGG TGG GAA CCA ACA AAG CTA AGC GAC AAA ACA GGT AAA 900

286 Leu Tyr Gly Trp Glu Pro Thr Lys Leu Ser Asp Lys Thr Gly Lys 300

901 CCG TCT ACG TCA TCA GAG GTA CTG GAA GAA CTC AAC TAC CCT TTA 945

301 Pro Ser Thr Ser Ser Glu Val Leu Glu Glu Leu Asn Tyr Pro Leu 315

946 GTG TCG TCT CTG GTC AAG TAT CAG AAG ATG GCT AAG CTG ATG CAG 990

316 Val Ser Ser Leu Val Lys Tyr Gln Lys Met Ala Lys Leu Met Gln 330

991 TTC ATA CCT CAG ATA CAA GCT AAC CTA GAC GGT AAC ATC CTT AGA 1035

331 Phe Ile Pro Gln Ile Gln Ala Asn Leu Asp Gly Asn Ile Leu Arg 345

1036 CCT AGC TAC AAC CAG TCT GCT ACA CGC ACG ACA CGA CTA TCA TCA 1080

346 Pro Ser Tyr Asn Gln Ser Ala Thr Arg Thr Thr Arg Leu Ser Ser 360

5’ss

1081 TCT AAG CCT TAA CAT GTA GGG CTT GTA AAA CCT TGT GAA CTC AGG 1125

361 Ser Lys Pro 375

1126 GAA AGC AAA GAC CTT GTA ACC CTG AGC GAA GCT CTG CCC ATA TCT 1170

376 390

1171 ACC TAT AGG AGG TAC ACG ATG GAC GAT AAA CTG AAG GAA CTC TTT 1215

391 Met Asp Asp Lys Leu Lys Glu Leu Phe 405

**RBS**

1216 TTA ACT ACT GAC CTT ACG TAC TAC CAG ATG GCA TAT GCG TTA GGT 1260

406 Leu Thr Thr Asp Leu Thr Tyr Tyr Gln Met Ala Tyr Ala Leu Gly 420

1261 ACA ACG TAC AAA GTA GTT CAT GGA CAT CTT ACT AAG TTG TTC AGC 1305

421 Thr Thr Tyr Lys Val Val His Gly His Leu Thr Lys Leu Phe Ser 435

1306 AAA GAA GAG AGA AAG GCT AGG ACA TCA CGT ATG TTA CGT CTT GCT 1350

436 Lys Glu Glu Arg Lys Ala Arg Thr Ser Arg Met Leu Arg Leu Ala 450

1351 CGT ACA GGT GAA AAG AAC CCC ATG TAC GGG ACA GAA AGT AAC AAA 1395

451 Arg Thr Gly Glu Lys Asn Pro Met Tyr Gly Thr Glu Ser Asn Lys 465

1396 CGG TTA GAC GTT ATC GCT GAT GGA CGC GGT TAC CTT ATG GTT CGT 1440

466 Arg Leu Asp Val Ile Ala Asp Gly Arg Gly Tyr Leu Met Val Arg 480

1441 AAA CCT ACC TGG TAC ACG GGA CGT AAA GGA AGT CGT CAT GTT TTT 1485

481 Lys Pro Thr Trp Tyr Thr Gly Arg Lys Gly Ser Arg His Val Phe 495

1486 CAA CAT CAC GTT GTT ATG TGT GAA GCG TTG GGT TTA ACA CAA GTA 1530

496 Gln His His Val Val Met Cys Glu Ala Leu Gly Leu Thr Gln Val 510

1531 CCT GAC GGG TTC CAT GTA CAT CAC GTA GAT GGT GAT AAG ACA AAC 1575

511 Pro Asp Gly Phe His Val His His Val Asp Gly Asp Lys Thr Asn 525

1576 AAC AAC ATC AAC AAC TTA GCC TTA CTA TGT GAC TCA GCG CAC AGT 1620

526 Asn Asn Ile Asn Asn Leu Ala Leu Leu Cys Asp Ser Ala His Ser 540

1621 AGA CTT CAT AGC AGA GAA CGT GCA ACG ACT ATC TCG AAA GAG AGT 1665

541 Arg Leu His Ser Arg Glu Arg Ala Thr Thr Ile Ser Lys Glu Ser 555

1666 AGG GTC TAG TAA ACC CGA AGC GCA AGG GAC ACA TCG AGA TAA CAT 1710

556 Arg Val End 570

1711 CTG GTG TGT CAT GAT ATA GTC TAC TCC TCG TGG GAA CAC GAG GTA 1755

571 585

3’ss

1756 TAA AGG AAC ATC CAA CAG ATA CCG TCA CGA GAC GAA CAC GGC AAA 1800

586 Asn Ile Gln Gln Ile Pro Ser Arg Asp Glu His Gly Lys 600

1801 GAA CTA CGC TCG TTG TTC TCT GCT CCT CCG GGT TGG TCG TTG GTT 1845

601 Glu Leu Arg Ser Leu Phe Ser Ala Pro Pro Gly Trp Ser Leu Val 615

1846 GTC GGT GAT CAG TCT GGT TTC CAG CTA CGT ATC ATG GCA GCG TAC 1890

616 Val Gly Asp Gln Ser Gly Phe Gln Leu Arg Ile Met Ala Ala Tyr 630

1891 ATG GAG ATG TAC TAC GAT GAA CCC CGA CTG TCT AAG GTG TTT GTC 1935

631 Met Glu Met Tyr Tyr Asp Glu Pro Arg Leu Ser Lys Val Phe Val 645

1936 GAT GGT GAA GAT GTT CAC CAG TTC TTT GCT GAC ATC TAC GGT ATC 1980

646 Asp Gly Glu Asp Val His Gln Phe Phe Ala Asp Ile Tyr Gly Ile 660

1981 GAT AGA AAG ATA GCC AAG AAC GTA ACC TTC GGT TAT GCG TTT GGT 2025

661 Asp Arg Lys Ile Ala Lys Asn Val Thr Phe Gly Tyr Ala Phe Gly 675

2026 GCT GGT GCT ACT AAG ATG GCG GCT ACT GCC TCA CGT GGT GGC TCT 2070

676 Ala Gly Ala Thr Lys Met Ala Ala Thr Ala Ser Arg Gly Gly Ser 690

2071 GTC GTG CCT GTG TCT ACT ATC AAA GGT GCG CTG TCT TCA CTG CAA 2115

691 Val Val Pro Val Ser Thr Ile Lys Gly Ala Leu Ser Ser Leu Gln 705

2116 GAT AGG CTA CCT GCA CTA CCA GCA CTT AAG ACA CTT GTT GTC GAA 2160

706 Asp Arg Leu Pro Ala Leu Pro Ala Leu Lys Thr Leu Val Val Glu 720

2161 CAT GCA CGA GAT AAC GGG GGT GTG TTC CAT GAC TTA CTA GGA CAA 2205

721 His Ala Arg Asp Asn Gly Gly Val Phe His Asp Leu Leu Gly Gln 735

2206 CGA TAT GTA GTT CCT GAG CTT CTG TCT AAG AAC AAA AGT GAA CGC 2250

736 Arg Tyr Val Val Pro Glu Leu Leu Ser Lys Asn Lys Ser Glu Arg 750

2251 GCT GCT GGT GAA CGT AGA GCG TTT AAC TAC TGG GTT CAA GGT TTT 2295

751 Ala Ala Gly Glu Arg Arg Ala Phe Asn Tyr Trp Val Gln Gly Phe 765

2296 GAA GCT ACA GCG TTT AGG TAC TTA CAG CTT AAA GCT AGA CCA GTA 2340

766 Glu Ala Thr Ala Phe Arg Tyr Leu Gln Leu Lys Ala Arg Pro Val 780

2341 CAG TTT GAG TAC GGT GCT AAG TTA GCG TTT GTT GTA CAT GAT GAG 2385

781 Gln Phe Glu Tyr Gly Ala Lys Leu Ala Phe Val Val His Asp Glu 795

2386 GTG GGT TAC TTA TGT CAG AGC GTG GTA GCG GAA GAG TTT GCT AAA 2430

796 Val Gly Tyr Leu Cys Gln Ser Val Val Ala Glu Glu Phe Ala Lys 810

2431 CAG ATG ACG AGC ATC TAT ACA ACT CGT GAA GTG TTC CCG TCT GAC 2475

811 Gln Met Thr Ser Ile Tyr Thr Thr Arg Glu Val Phe Pro Ser Asp 825

2476 ATG ACA AGT GGT TTG ACA CTA GAA GCT GAG TTC CAA GTA GGA CAG 2520

826 Met Thr Ser Gly Leu Thr Leu Glu Ala Glu Phe Gln Val Gly Gln 840

2521 ACA TGG CTA GAG GCG AAG TAG 2541

841 Thr Trp Leu Glu Ala Lys End
