# Supplementary figures and images for: I-PfoP3I: A Novel Nicking HNH Homing Endonuclease Encoded in the Group I Intron of the DNA Polymerase Gene in Phormidium foveolarum Phage Pf-WMP3
Source: PLoS One. 2012 Aug 27;7(8):e43738. doi: 10.1371/journal.pone.0043738 (PMC3428280; doi:10.1371/journal.pone.0043738)

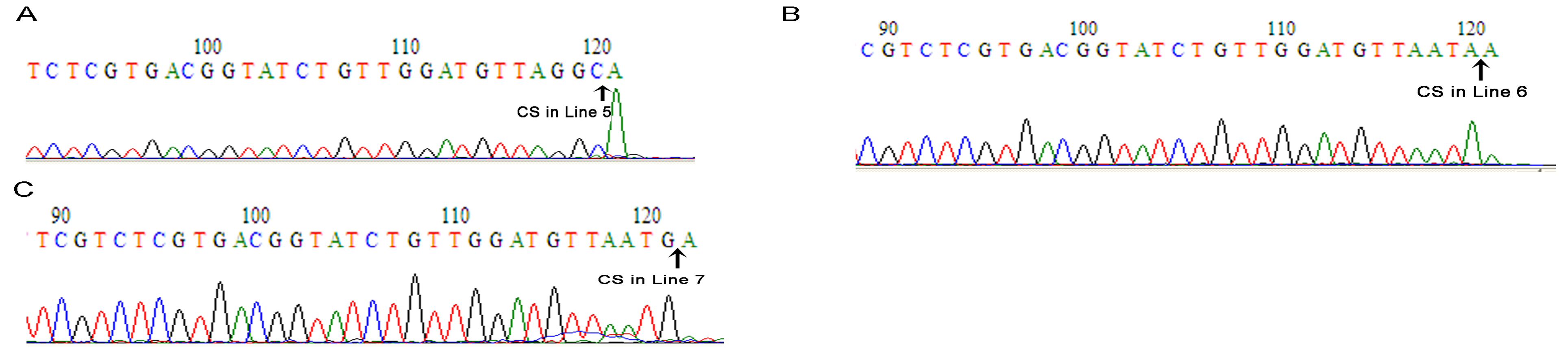

Supplement: Figure S2 — Determination of the cleavage sites (CS) by direct DNA sequencing of cleaved PCR products of Pf-WMP3 DNAP gene with deletions ( Figure 9 , Line 5, 6, 7) using reverse primers P3r1. The open arrows indicate cleavage position of I-PfoP3I. (TIF) [file pone.0043738.s002.tif]
