# Supplementary material for: I-PfoP3I: A Novel Nicking HNH Homing Endonuclease Encoded in the Group I Intron of the DNA Polymerase Gene in Phormidium foveolarum Phage Pf-WMP3
Source: PLoS One. 2012 Aug 27;7(8):e43738. doi: 10.1371/journal.pone.0043738 (PMC3428280; doi:10.1371/journal.pone.0043738)
Supplement: Table S1 — Oligonucleotides used to determine the I-PfoP3I recognition site boundaries contain variants of the wild-type intronless sequence. (DOC) [file pone.0043738.s003.doc]

| 1f | 5′- GGCTAAGCTGATGCAACTATCATCATCTAA; |
| --- | --- |
| 2f | 5′- TACACGCACGACACGCATCATCTAAGCCTA; |
| 3f | 5′- GCACGACACGACTATTCTAAGCCTAACATC; |
| 4f | 5′- ACGACACGACTATCATAAGCCTAACATCCA; |
| 5f | 5′- ACGCACGACACGACTGCCTAACATCCAACA; |
| 6f | 5′- GCACGACACGACTATTAACATCCAACAGAT; |
| 7f | 5′- CGACACGACTATCATTAACATCCAACAGAT; |
| 8f | 5′- ACACGACTATCATGAACATCCAACAGATAC; |
| 9f | 5′- CGACACGACTATCATGTACATCCAACAGATAC; |
| 10f | 5′- CACGACTATCATGTCCATCCAACAGATACC; |
| 11f | 5′- GACACGACTATCATGTCACTCCAACAGATACCGT; |
| 12f | 5′- GCACGACACGACTATCAACAGATACCGTCA; |
| 13f | 5′- CATCATCTAAGCCTACAACAGATACCGTCA; |
| 14f | 5′- TCTAAGCCTAACATCACCGTCACGAGACGA; |
| 15f | 5′- CTAAGCCTAACATCCCGTCACGAGACGAAC; |
| 16f | 5′- TAAGCCTAACATCCACGTCACGAGACGAAC; |
| 17f | 5′- AAGCCTAACATCCAAGTCACGAGACGAACA; |
| 18f | 5′- AGCCTAACATCCAACGATACCGTCACGAGA; |
| 19f | 5′- GCCTAACATCCAACATCACGAGACGAACAC; |
| 20f | 5′-CGACACGACTATTAACATCCAACGATACCGTCACG; |
| 21f | 5′-ACGACACGACTACTAACATCCAACAATACCGTCACGA; |
| 22f | 5′-CACGACACGACTACTAACATCCAACAGTACCGTCACGAG; |
| 23f | 5′-CACGACACGACTCCTAACATCCAACAATACCGTCACGAG; |
| 24f | 5′-CACGACACGACTCCTAACATCCAACAGTACCGTCACGAG; |
| 25f | 5′-ACGCACGACACGACTTACCGTCACGAGACG; |
| 1r | 5′- TTAGATGATGATAGTTGCATCAGCTTAGCC; |
| 2r | 5′- TAGGCTTAGATGATGCGTGTCGTGCGTGTA; |
| 3r | 5′- GATGTTAGGCTTAGAATAGTCGTGTCGTGC; |
| 4r | 5′- TGGATGTTAGGCTTATGATAGTCGTGTCGT; |
| 5r | 5′- TGTTGGATGTTAGGCAGTCGTGTCGTGCGT; |
| 6r | 5′- ATCTGTTGGATGTTAATAGTCGTGTCGTGC; |
| 7r | 5′- ATCTGTTGGATGTTAATGATAGTCGTGTCG; |
| 8r | 5′- GTATCTGTTGGATGTTCATGATAGTCGTGT; |
| 9r | 5′- GTATCTGTTGGATGTACATGATAGTCGTGTCG; |
| 10r | 5′- GGTATCTGTTGGATGGACATGATAGTCGTG; |
| 11r | 5′- ACGGTATCTGTTGGAGTGACATGATAGTCGTGTC; |
| 12r | 5′- TGACGGTATCTGTTGATAGTCGTGTCGTGC; |
| 13r | 5′- TGACGGTATCTGTTGTAGGCTTAGATGATG; |
| 14r | 5′- TCGTCTCGTGACGGTGATGTTAGGCTTAGA; |
| 15r | 5′- GTTCGTCTCGTGACGGGATGTTAGGCTTAG; |
| 16r | 5′- GTTCGTCTCGTGACGTGGATGTTAGGCTTA; |
| 17r | 5′- TGTTCGTCTCGTGACTTGGATGTTAGGCTT; |
| 18r | 5′- TCTCGTGACGGTATCGTTGGATGTTAGGCT; |
| 19r | 5′- GTGTTCGTCTCGTGATGTTGGATGTTAGGC; |
| 20r | 5′- CGTGACGGTATCGTTGGATGTTAATAGTCGTGTCG; |
| 21r | 5′- TCGTGACGGTATTGTTGGATGTTAGTAGTCGTGTCGT; |
| 22r | 5′- CTCGTGACGGTACTGTTGGATGTTAGTAGTCGTGTCGTG; |
| 23r | 5′- CTCGTGACGGTATTGTTGGATGTTAGGAGTCGTGTCGTG; |
| 24r | 5′- CTCGTGACGGTACTGTTGGATGTTAGGAGTCGTGTCGTG; |
| 25r | 5′- CGTCTCGTGACGGTAAGTCGTGTCGTGCGT. |
